# Treatment of Ingrowing Nail

**Published:** 1888-12

**Authors:** 


### TREATMENT OF INGROWING NAIL.

The following procedure for removal of ingrowing toe-nail, has been employed with excellent results in many cases. After thorough cleansing of the nail a solution of gutta percha 10 parts, in 80 parts of chloroform, is applied with a brush to the interstices between the nail and the granulations. This is repeated several times on the first day, and subsequently at longer intervals. By exercise of care and patience it will be found that the nail is gradually lifted from the underlying parts, and can then be removed without pain with the scissors. If a properly-fitted shoe is worn no recurrences need to be apprehended. The solution applied in this manner exerts a double effect, the chloroform is anæsthetic, and the gutta percha acts mechanically, forcing its way between the granulations and the nail, and finally liberating it from its abnormal position.

